# Transcription and post-translational mechanisms: dual regulation of adiponectin-mediated Occludin expression in diabetes

**DOI:** 10.1186/s13578-024-01306-5

**Published:** 2024-10-01

**Authors:** Yanru Duan, Demin Liu, Huahui Yu, Shihan Zhang, Yihua Xia, Zhiyong Du, Yanwen Qin, Yajing Wang, Xinliang Ma, Huirong Liu, Yunhui Du

**Affiliations:** 1 Department of Physiology and Pathophysiology, School of Basic Medical Sciences, Capital Medical University, Beijing, 100069 People’s Republic of China; 2 Department of Cardiology, The Second Hospital of Hebei Medical University, Shijiazhuang, 050000 Hebei China; 3 Beijing Anzhen Hospital, Capital Medical University, Beijing Institute of Heart, Lung and Blood Vessel Diseases, Beijing, 100029 People’s Republic of China; 4 Medical Oncology Department, Pediatric Oncology Center, Beijing Children’s Hospital, Capital Medical University, National Center for Children’s Health, Beijing Key Laboratory of Pediatric Hematology Oncology, Key Laboratory of Major Diseases in Children, Ministry of Education, Beijing, 100045 People’s Republic of China; 5 Department of Cardiology, China-Japan Friendship Hospital, Beijing, 100029 China; 6 Department of Biomedical Engineering, University of Alabama at Birmingham, Birmingham, AL 35294 USA; 7 Department of Emergency Medicine, Thomas Jefferson University, Philadelphia, PA 19107 USA; 8 Beijing Key Laboratory of Metabolic Disturbance Related Cardiovascular Disease, Beijing, China

**Keywords:** Diabetes, Adiponectin, Occludin, Transcription, Post-translation modification

## Abstract

**Background:**

Occludin, a crucial component of tight junctions, has emerged as a promising biomarker for the diagnosis of acute ischemic disease, highlighting its significant potential in clinical applications. In the diabetes, Occludin serves as a downstream target gene intricately regulated by the adiponectin (APN) signaling pathway. However, the specific mechanism by which adiponectin regulates Occludin expression remains unclear.

**Methods and results:**

Endothelial-specific *Ocln* knockdown reduced APN-mediated blood flow recovery after femoral artery ligation and nullified APN's protection against high-fat diet (HFD)-triggered apoptosis and angiogenesis inhibition in vivo. Mechanically, we have meticulously elucidated APN's regulatory role in Occludin expression through a comprehensive analysis spanning transcriptional and post-translational dimensions. Foxo1 has been elucidated as a crucial transcriptional regulator of Occludin that is modulated by the APN/APPL1 signaling axis, as evidenced by validation through ChIP-qPCR assays and Western blot analysis. APN hindered Occludin degradation via the ubiquitin–proteasome pathway. Mass spectrometry analysis has recently uncovered a novel phosphorylation site, Tyr467, on Occludin. This site responds to APN, playing a crucial role in inhibiting Occludin ubiquitination by APN. The anti-apoptotic and pro-angiogenic effects of APN were attenuated in vitro and in vivo following Foxo1 knockdown or expression of a non-phosphorylatable mutant, OccludinY467A. Clinically, elevated plasma concentrations of Occludin were observed in patients with diabetes. A significant negative correlation was found between Occludin levels and APN concentrations.

**Conclusion:**

Our study proposes that APN modulates Occludin expression through mechanisms involving both transcriptional and post-translational interactions, thereby conferring a protective effect on endothelial integrity within diabetic vasculature.

**Graphical abstract:**

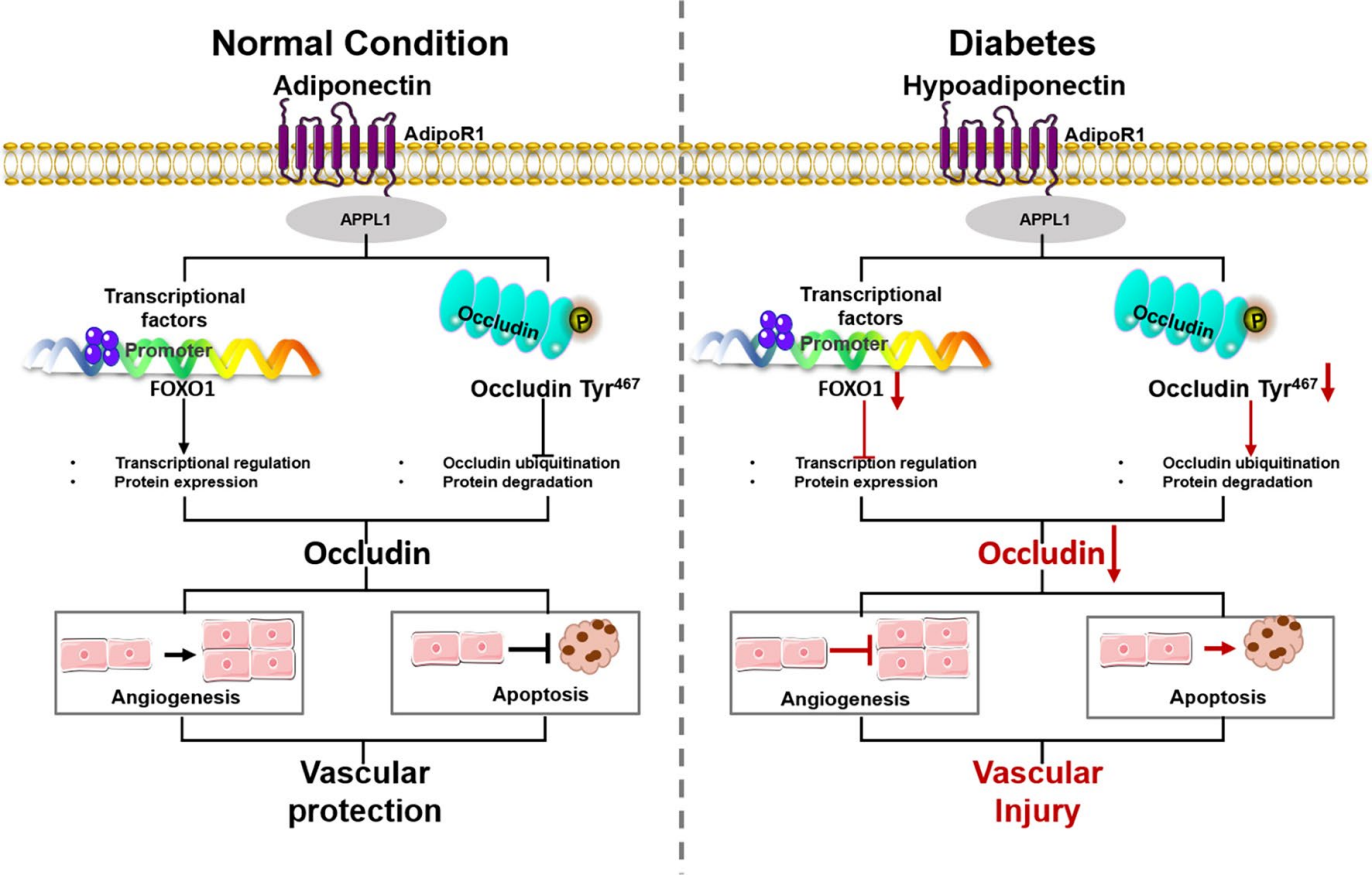

**Supplementary Information:**

The online version contains supplementary material available at 10.1186/s13578-024-01306-5.

## Introduction

Vascular injury stands out as a crucial and initiating factor in the pathogenesis of cardiovascular diseases in individuals with diabetes [1]. However, current understanding of the protective mechanisms against diabetic vascular injury remains limited, necessitating extensive research for deeper insight and potential therapeutic targets. Adiponectin (APN), an intrinsic biologically active polypeptide, has emerged as a focal point of extensive scientific inquiry [2]. A wealth of experimental evidence substantiates the role of APN in markedly ameliorating the pathological alterations characteristic of diabetic vascular endothelium [3–5]. Concurrently, APN plays a pivotal role in orchestrating the regulatory network of vascular protective genes, thus conferring a substantial protective effect on the vasculature [6–10]. However, the complex mechanisms by which APN influences the expression of these vascular protective genes require further elucidation, representing a crucial area for advanced research.

Occludin, recognized as a key protein integral to the structure of tight junctions, plays a crucial role in regulating cellular permeability. It achieves this by orchestrating intercellular connectivity, thus serving an essential function in maintaining barrier integrity [11, 12]. Alterations in Occludin expression are critically implicated in the pathogenesis of vascular endothelial dysfunction [13]. Notably, in individuals with diabetes, there is a notable reduction in Occludin levels within retinal vascular endothelial cells. This decrease is associated with increased vascular permeability, dysregulated angiogenesis, and heightened inflammatory responses. These observations suggest that decreased Occludin expression may contribute to the development of vascular dysfunction in diabetes [14, 15]. In our prior investigations, we have elucidated that Occludin acts as a downstream effector molecule in the APN/APPL1 signaling pathway, exerting a protective influence on the vascular endothelium in diabetic conditions. At the cellular level, gene-level suppression of *Ocln* has been demonstrated to counteract the protective effects of APN against endothelial cell apoptosis and increased permeability associated with diabetes. This underscores the potentially pivotal role of Occludin in APN-mediated endothelial protection [16]. Nonetheless, the intricate mechanisms by which the APN/APPL1 axis modulates Occludin require further detailed elucidation.

In our study, we observed that genetic suppression of *Ocln*, both in vivo and in vitro, abolished the beneficial effects of APN on diabetes-induced endothelial cell apoptosis and angiogenesis. These findings underscore the crucial role of Occludin in mediating the vascular protective effects of APN. Furthermore, our studies identified Foxo1, regulated by APN, as a transcription factor that governs the expression of Occludin. Concurrently, it was also established that APN impedes the degradation of Occludin via the ubiquitin–proteasome pathway (UPP), with phosphorylation at the Tyr467 site of Occludin playing a critical role in mediating APN's inhibitory effect on Occludin ubiquitination. Functionally, the pathologic and therapeutic significance of APN-mediated transcriptional regulation, post-translational modifications, and resulting protein expression of Occludin were evidenced crucially in the context of diabetic vascular protection.

## Materials and methods

### Human participants

Forty five patients with type 2 diabetes (male: 28, female: 17) and 20 healthy control individuals (male: 12, female: 8) were recruited from Beijing Anzhen Hospital. The clinical profiles of the diabetic patients are detailed in Supplementary Table 1. The study protocol received approval from the Ethics Committee of Beijing Anzhen Hospital (No. 2017005) and adhered to the principles of the Declaration of Helsinki. Written and verbal informed consents were obtained from all participants. Venous blood samples were collected upon admission without the use of anticoagulants. After centrifugation at 4 °C, plasma was promptly separated and stored at − 80 °C for subsequent analysis. To ensure data integrity, we excluded patients with a history of stroke, type 1 diabetes mellitus (T1DM), valvular heart disease, severe cardiovascular disease (CVD) classified as level III or IV by New York Heart Association criteria, significant hepatic or renal insufficiency, recent infectious diseases within the past two months, active liver disease, hemodialysis, malignancies, pregnancy, or hyperthyroidism.

### Animal models

All experiments in this study were conducted in compliance with the NIH Guidelines for the Use of Laboratory Animals and were approved by the Capital Medical University Committee on Animal Care. APPL1 knockout mice (male APPL1^−/−^, 8 weeks old) and C57BL/6J mice were used in the study. The mice were randomly assigned to either a high-fat diet (HFD) (60% kcal fat, D12492i; Research Diets Inc.) or a standard diet (ND, D12450Bi) for 12 weeks to induce type 2 diabetes, as determined by fasting blood glucose levels (Figure S1). After 12 weeks, the mice were administered either vehicle or globular adiponectin treatment (0.25 μg/g/day intraperitoneally, PeproTech, Cranbury, NJ, United States) via a miniosmotic pump (ALZET, DURECT Corp, Cupertino, CA, United States) for an additional two weeks.

### Packaging of ECAAV9-shOcln/ECAAV9-shFoxo1/ECAAV9-hOcln-Y467A

Packaging of ECAAV9-sh*Ocln*/ECAAV9-sh*Foxo1*/ECAAV9-h*Ocln*-Y467A: pAAV-*Ocln/Foxo1* shRNAs were designed as follows: *Ocln* siRNA: 5ʹ-CCAUGGCAUACUCUUCCAATT-3ʹ; *Foxo1* siRNA: 5ʹ-GAGCGUGCCCUACUUCAAGTT-3ʹ. The pAAV-h*Ocln*-Y467A construct was based on NM_002538.3. The pECAAV9 helper plasmid (for packaging with SIGYPLP peptide) was developed by Beijing Likely Biotechnology Co., Ltd. To prepare retargeted AAV vectors, 24 plates of 293 cells at 80% confluence were co-transfected with 50 µg of pAAV-*Ocln*/Foxo1 shRNAs, adenovirus helper plasmid (pHelper), and pECAAV9 at a mass ratio of 2:2:1. After 60 h, cells were harvested, pelleted by centrifugation, and resuspended in 150 mM NaCl, 50 mM Tris–HCl (pH 8.5). The cells underwent multiple freeze–thaw cycles. Cell debris was cleared by centrifugation and filtration through a 0.45 µm PVDF filter. The viral supernatant was purified using ViraBind™ AAV Purification Kits (Cell BioLabs, VPK-141) and finally eluted with 1 ml of buffer. Eight-week-old C57BL/6J mice were administered a single dose of ECAAV9-scramble, ECAAV9-sh*Ocln*, or ECAAV9-shFoxo1 (5 × 10^11^ GC/mouse) via the retro-orbital route.

### Cell apoptosis assay

Cell apoptosis was assessed using flow cytometry with a FITC-Annexin V Apoptosis Detection Kit (Yeasen Biotech). Cells were harvested using mild trypsin digestion. Double staining was performed with FITC-Annexin V and propidium iodide following the manufacturer’s protocol, and analysis was conducted using Gallios flow cytometry (Beckman Coulter). Data acquisition was performed using Kaluza for Gallios software (Beckman Coulter), collecting a minimum of 10,000 events per analysis. We categorized cells into living, dead, early apoptotic, and late apoptotic stages. The late apoptotic cells served as the target for our comparative analysis.

### In vitro phosphorylation assays and mass spectrometry analysis

Phosphorylation reactions were conducted in a 50-μL reaction volume containing 1 × kinase buffer, 0.2 mmol/L ATP, 250 ng APN, 900 ng Occludin (Proteintech, Ag26173), and ddH_2_O. Reactions were started for 40 min at 30 °C and were terminated by the addition of 6 × sample loading dye (10 μL). Samples were heated for 5 min at 95 °C. Then, each reaction mixture was loaded onto 10% Tris–glycine gel. After electrophoresis followed by Coomassie bright blue stain, protein bands of interest were excised from the gel slab and processed for in-gel digestion by trypsin. Where specified, recovered tryptic peptides were analyzed. Recovered peptides from each protein were analyzed using nanoelectrospray tandem mass spectrometry on a Q-TOF hybrid mass spectrometer (QSTAR-Pulsar, Applied Biosystems/Sciex and Bruker-Daltonics AutoFlex TOF-TOF LIFT). The mass profiles of tryptic peptides were subsequently assessed by querying the protein sequence databases (NCBI Nonredundant Protein Database).

### Small interfering RNA transfection

siRNA duplex oligonucleotides were designed to target specific gene sequences, effectively silencing the expression of APPL1 and Foxo1 genes. Human umbilical vein endothelial cells (HUVEC) were transfected using the Lipofectamine^®^ 3000 Transfection Kit (Thermo Fisher Scientific, Inc) following the manufacturer's instructions, employing siRNA duplexes against APPL1 (5ʹ-UCUCACCUGACUUCGAAACUdTdT-3ʹ) and Foxo1 (5ʹ-GAGCGUGCCCUACUUCAAGTT-3ʹ), as well as universal control oligonucleotides (AllStars, Westerville, OH, USA). Cells were initially seeded in six-well plates and allowed to reach 80% confluence before siRNA transfection, with a final concentration of 50 nM applied to each well.

### Chromatin immunoprecipitation-qPCR

Chromatin immunoprecipitation (ChIP) assays were conducted using an EZ-ChIP kit (Abcam, Cambridge, MA, USA) following the manufacturer's protocol. Briefly, three biological replicates of HUVECs were cross-linked with 1% paraformaldehyde and subsequently lysed in cold phosphate-buffered saline (PBS) containing a protease inhibitor cocktail. Chromatin was fragmented by sonication using a Covaris 220 instrument, with 10-s pulses interspersed with 30-s pauses, repeated 10 times. Immunoprecipitation was carried out overnight at 4 °C using 1 μg of anti-Foxo1 antibody (#2880, CST) and IgG control antibody (#2729, CST). Each immunoprecipitated sample was incubated with protein G agarose beads for 3 h at 4 °C. Following pull-down, beads were washed five times to remove non-specific binding. The immunocomplexes were then eluted and subjected to reverse cross-linking, proteinase K digestion, and DNA precipitation. The recovered DNA was analyzed by quantitative PCR (qPCR). Primers targeting the promoter regions of the *Ocln* genes were utilized to amplify both input and immunoprecipitated DNA (5ʹ-GAAGTGGGTGGGATTGGATAG-3ʹ). All experiments were conducted in triplicate from at least two independent experiments, and the data were normalized to percent input.

### ChIP-qPCR data analysis

Normalize each ChIP DNA fraction's Ct value to the Input DNA fraction's Ct value for the same qPCR assay (ΔCt) to account for variations in chromatin sample preparation. Calculate the %Input for each ChIP fraction using the formula: %Input = 2^(CtInput—CtChIP) × Fd × 100%. Here, Fd represents the Input dilution factor. In our study, a 100 μL sonicated sample was used for ChIP, while a 20 μL sonicated sample served as Input, resulting in Fd = 1/5. To determine Fold Enrichment, use the formula: Fold Enrichment = [% (ChIP/Input)]/[% (IgG/Input)]. The fold enrichment value for the Vehicle group is standardized to “1”, with other groups being compared relative to the Vehicle group.

### Transcription factor (TF) profiling array

The Transcription Factor Activation Profiling Plate Array II (Signosis, Sunnyvale, CA, USA) was utilized to assess the activity of transcription factors in HUVEC cells following the manufacturer's guidelines. Nuclear proteins from HUVEC cells were extracted using a Nuclear Extraction Kit (Signosis, Sunnyvale, CA, USA). Biotin-labeled probes, designed based on consensus sequences of transcription factor DNA-binding sites, were combined with 15 μg of nuclear protein extract to form transcription factor/probe complexes. The bound probes were then separated from the complex and hybridized to a plate pre-coated with sequences complementary to the probes. The captured DNA probe was detected using streptavidin-HRP, and the signal intensity was quantified using a microplate luminometer. Relative gene expression levels were determined using the ΔΔCT method, normalizing to the average expression level of five housekeeping genes.

### RT^2^ Profiler™ PCR array for human transcription factors and quantitative real time-PCR (qRT-PCR) analysis

The Human Transcription Factors Array (Qiagen, USA) was utilized to assess the mRNA levels of human transcription factors in HUVEC cells in accordance with the manufacturer's instructions. Total RNA was extracted using the Trizol reagent method (Invitrogen) and employed for first-strand cDNA synthesis. qRT-PCR was conducted using RT^2^ SYBR Green Mastermix (PARN-026Z, QIAGEN) on a 7500 Real-Time PCR system (Thermo Fisher Scientific, Inc). The PCR protocol consisted of an initial denaturation step at 95 °C for 10 min, followed by 40 cycles of denaturation at 95 °C for 15 s and annealing/extension at 60 °C for 1 min. Gene expression levels were determined based on the CT values, and fold-changes in expression were calculated using the 2^−ΔΔCT^ method. All samples were analyzed in triplicate.

### Immunoprecipitation and Western blot analysis

Cells were rinsed once with PBS and subsequently lysed using cold 1 × lysis buffer supplemented with a protease inhibitor cocktail (Thermo Fisher Scientific, 78438). For immunoprecipitation, 1 μg of the appropriate control IgG was added to the cell lysate, followed by 20 μL of resuspended Protein A/G Plus-Agarose beads. The mixture was incubated at 4 °C for 30 min. The cleared lysate was then combined with normal immunoglobulin G (IgG) and anti-Occludin primary antibodies (Invitrogen, #71-1500), along with 15 μL of pre-washed Protein A beads, and incubated overnight at 4 °C. The immunoprecipitated proteins were subsequently released from the beads using an elution buffer. Samples were boiled, separated by SDS-PAGE, and analyzed via Western blotting.

Mouse aortic vascular tissues and cells were harvested and lysed to extract total protein. Protein concentrations were determined using the BCA Protein Assay Kit (Thermo Fisher Scientific, Inc. 23227). The total proteins were separated by gel electrophoresis and transferred onto a poly-vinylidene fluoride (PVDF) membrane. The membranes were then blocked with 5% nonfat milk for 1 h, followed by overnight incubation at 4 °C with primary antibodies. After washing, the membranes were incubated with HRP-conjugated secondary antibodies at room temperature for 1 h. Protein bands were visualized using the BioRad Imaging System. Antibodies against GAPDH (#5174), Phospho (#2981), and HA (#5017) were obtained from Cell Signaling Technology (Beverly, MA), while the antibody against Flag (Cat. No: F9291) was purchased from Sigma.

### Confocal microscopic analysis

Endothelial cells were fixed in paraformaldehyde/PBS for 15 min and then washed with PBS. The cells were incubated with primary antibodies (rabbit anti-Occludin or mouse anti-LAMP2 antibody) at a 1:200 dilution. Subsequently, they were treated with either tetramethyl rhodamine (TRITC)-conjugated anti-mouse IgG or FITC-conjugated anti-rabbit IgG at a 1:100 dilution. After another wash with PBS, coverslips were mounted using an anti-fade solution (KPL, Gaithersburg, MD). Negative controls, which lacked primary antibodies, were also processed similarly. Fluorescent images were captured using Fluoview software (Olympus) on an FV3000 confocal microscope (Olympus, Tokyo, Japan).

### Enzyme-linked immunosorbent assay (ELISA)

Blood samples were collected at the time of inclusion to measure Adiponectin and Occludin levels. Plasma samples obtained at enrolment were promptly stored at − 80 °C in a dedicated biologic resource center. The levels of plasma Adiponectin and Occludin were quantified using commercial ELISA kits (Cat #SEA605Mu, Cat#SEC228Hu, Cloud-Clone Corp) following the manufacturer's protocols. All sample preparations, as well as the utilization of reagents and buffers, strictly conformed to the manufacturer’s guidelines.

### Mouse hindlimb ischemia model

Hindlimb ischemia (HLI) was induced by ligating the left femoral artery, starting from the distal point of its bifurcation down to the saphenous artery. Blood flow in the hindlimbs was assessed both before and immediately after ligation using laser Doppler flowmetry (LDF; PeriCam PSI). Blood flow was assessed and analyzed in a blinded manner. Mice exhibiting a reduction in blood flow of no less than 50% in the left hindlimb post-ligation were included in this study. For the mice with a successful ligation, another four measurements of blood flow were performed at day 0, 4, and 7.

### Statistical analyses

Quantitative results are presented as mean ± SEM or median with interquartile range. Comparisons between two groups were assessed using an unpaired t-test. For comparisons involving more than two groups, one-way ANOVA or two-way ANOVA was employed, followed by post hoc analysis using the Tukey test. Prior to conducting parametric tests such as t-tests and ANOVA, assumptions of equal variance and normality were checked. Equal variance was examined using Bartlett’s test, while normality was assessed using the Kolmogorov–Smirnov test. In cases where data exhibited a normal distribution but had unequal variances, the Welch t-test or Brown-Forsythe and Welch ANOVA were utilized. For data that did not meet the normality assumption, the Kruskal–Wallis nonparametric test was applied. Statistical analyses were performed using GraphPad Prism 8.0 (GraphPad Software, Inc, San Diego, CA, USA) and SPSS 24.0 (SPSS, Inc, Chicago, IL, USA). A p-value of less than 0.05 was considered statistically significant.

## Results

### Occludin is essential in APN-mediated diabetic vascular protection

To elucidate the mechanism by which APN regulates the gene expression of *Ocln,* both in vivo and in vitro experiments were conducted independently to assess the mRNA and protein levels of Occludin. PCR analysis revealed that *Ocln* expression was suppressed in endothelial cells subjected to high glucose/high lipids (HG/HL) treatment, but was enhanced upon APN intervention, an effect mediated via the APPL1 pathway (Figure S2A). Mouse aortic tissues were harvested in vivo to investigate the mRNA expression levels of *Ocln*. The data suggest that in contrast to APN-treating, WT mice on a high fat diet inhibited the mRNA levels of *Ocln*. However, APN increases *Ocln* mRNA levels in an APPL1 dependent manner (Figure S2B). Furthermore, the protein levels of Occludin were notably reduced in mice fed a high-fat diet (HFD) compared to those on a normal diet (ND) among wild-type (WT) mice. Administration of APN led to an upregulation of Occludin expression in WT aortic tissue, an effect that was absent in APPL1KO mice (Figure S2C). In alignment with these in vivo observations, Occludin protein levels were significantly suppressed in endothelial cells (ECs) treated with HG/HL. APN treatment restored Occludin protein expression in cells transfected with scrambled siRNA, a restorative effect that was abolished in cells transfected with siAPPL1 (Figure S2D). These results imply that both HG/HL and APN affect the expression of Occludin from the transcription and translation levels.

**Fig. 1:**
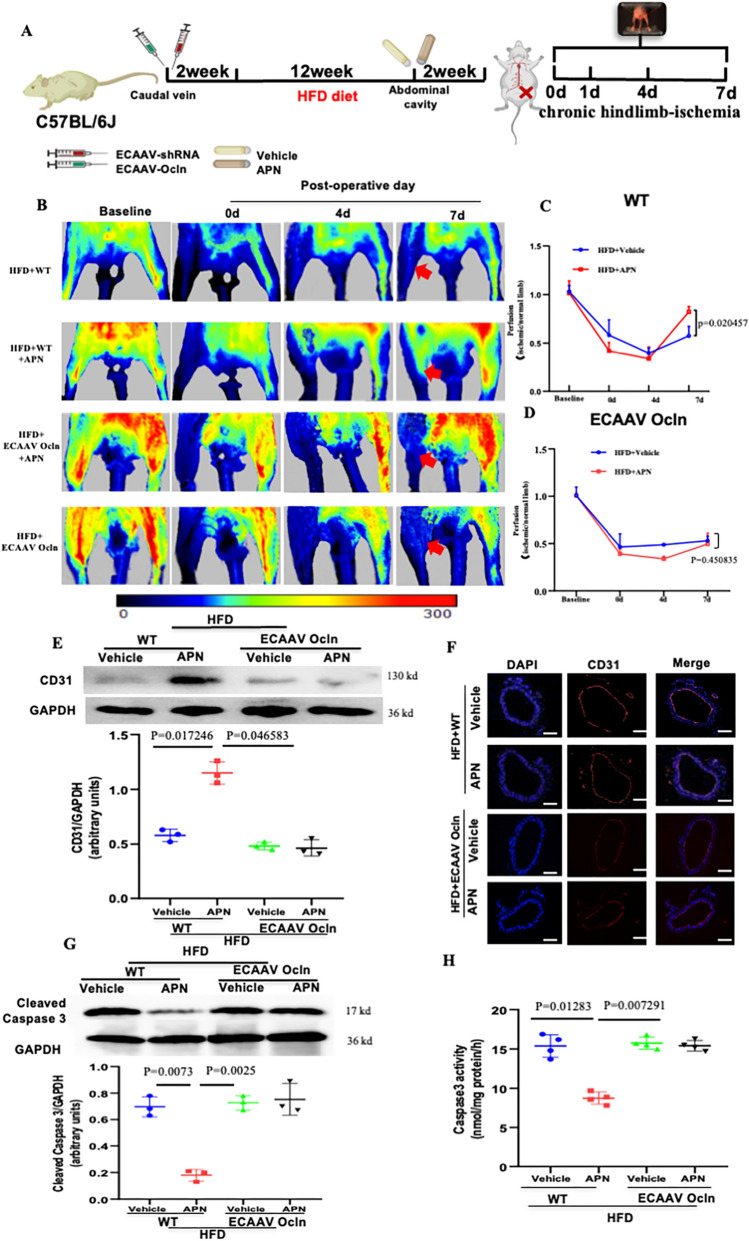
Occludin is extremely significant in APN-mediated diabetic vascular protection. **A** Schematic of animal model construction. **B** The blood flow of mice subjected to hindlimb ischemia was monitored at indicated time points in blinded fashion. **C**, **D** APN administration accelerated blood flow recovery in HFD mice, an effect significantly blunted in endothelial-specific knockdown *Ocln* mice. N = 6–8/group. Red arrow indicates blood flow in hindlimb. Data were analyzed by repeated-measures ANOVA (GraphPad Prism 8.0). **E** APN treatment inhibited the activation of CD31 expression in aortic tissues, an effect abolished by endothelial-specific Occludin knockdown. **F** Representative images of CD31 immunostaining in aorta localization. **G** APN attenuated apoptotic marker expression of cleaved caspase 3. The effect was significantly blunted by *Ocln* knockdown. **H** The activity of caspase 3 in aortic tissues of mouse. Data were analyzed by two-way analysis of variance. Comparisons between each group were made by post hoc analysis via Tukey test (GraphPad Prism 8.0). HFD indicates high-fat diet

Our previous study has demonstrated a pivotal role for Occludin in the amelioration of vascular injuries associated with diabetes, specifically those mediated by APN [16]. To investigate the role of Occludin in the vascular endothelium of diabetic models in vivo, both AAV9/shRNA-mediated EC-specific *Ocln* knockdown mice (Figure S3A, B) and AAV9/shRNA-mediated *Ocln* knockdown mice were sustained on either a ND or a HFD for a duration of 12 weeks. Twelve weeks subsequent to the initiation of the HFD, the animals underwent a procedure to induce chronic hindlimb ischemia. Immediately following the ischemic event, the mice received either a control treatment (vehicle) or APN. The assessment of blood flow was conducted using laser Doppler imaging (Fig. 1A). In control group, the administration of APN significantly enhanced the recovery of blood flow following femoral artery ligation. In contrast, in both the endothelial cell-specific *Ocln* knockdown mice and the general *Ocln* knockdown mice, APN administration did not yield an improvement in blood flow recovery (Fig. 1B–D; Figure S4). Simultaneously, the expression of CD31, a marker of angiogenic proteins, was analyzed in the muscles of hindlimbs undergoing ischemia through immunofluorescence staining. The results demonstrate that APN failed to promote the expression of CD31 in HFD-fed EC-specific *Ocln* knockdown mice implying a critical role for Occludin in APN-mediated angiogenesis within vascular endothelium (Figure S5). Concurrently, this observation was similarly noted in the aortic tissue specimens. APN treatment markedly enhanced CD31 expression, as evidenced by Western blot analysis (Fig. 1E) and immunofluorescence studies (Fig. 1F). However, these effects were nullified in cases of endothelial cell-specific knockdown of Occludin. Furthermore, our findings reveal that Occludin contributes to the anti-apoptotic response induced by APN in diabetic environments, evidenced by the analysis of cleaved caspase-3 expression (Fig. 1G) and the assessment of caspase-3 activity (Fig. 1H).

### Foxo1 is a transcriptional factor of Occludin mediated by APN

**Fig. 2:**
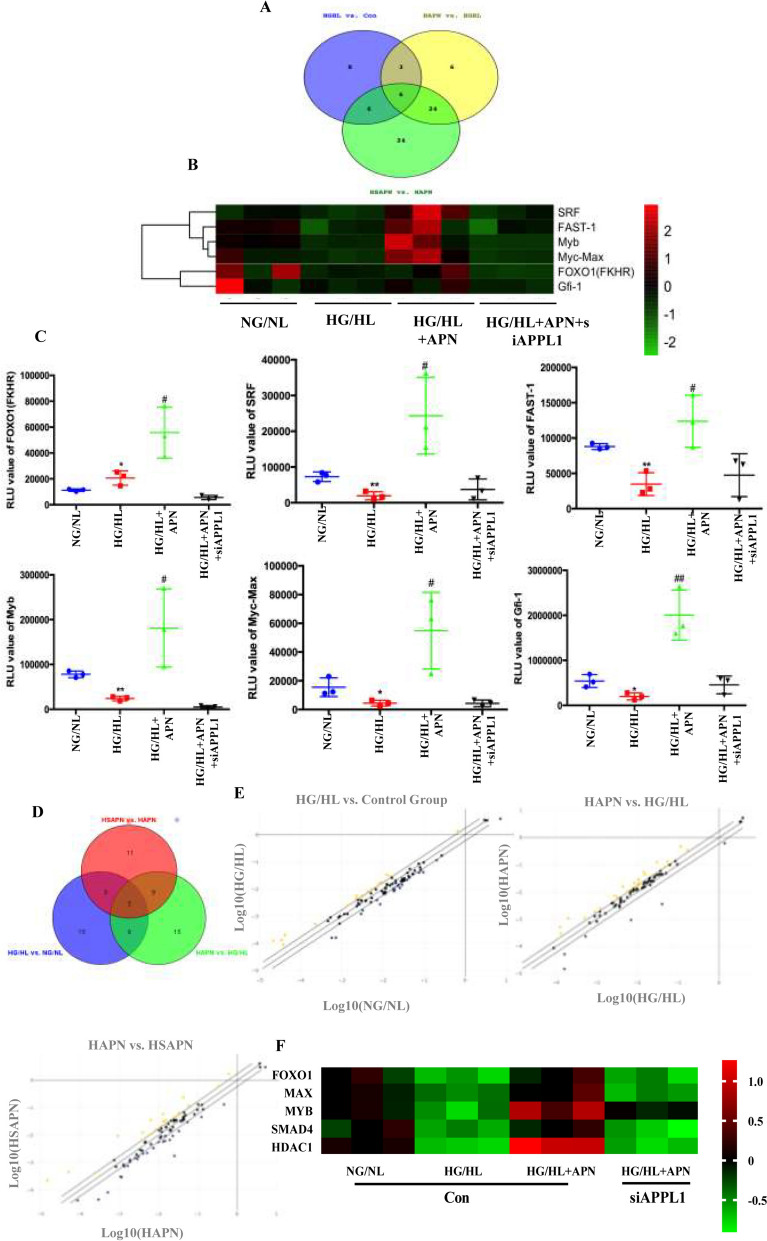
Alterations in the Expression Profile of Transcription Factors in APN-mediated, APPL1-dependent diabetic endothelium. **A** Analysis of comparative activity of transcription factors using Venn diagram. **B** Heatmap demonstrating the 6 upregulated transcription factors in HG/HL with APN in HUVECs by Transcription Factor (TF) Profiling Array. **C** 6 differential transcriptional factors involved in APN/APPL1 signaling pathway were identified using q-PCR. **D** Venn diagram analysis of comparative transcription factors expression. **E** Scatter plots showing differentially APPL1-related, APN-regulated transcription factors identified by the Human Transcription factor RT^2^ profiler PCR array. Shown are the relative changes in gene expression accordingly to 2^−ΔΔCT^ method with normalization to the average expression level of 5 housekeeping genes (Actb, B2m, Gapdh, Gusb, and Hsp90ab1). Yellow circles: genes upregulated > 1.2-fold; blue circles: genes downregulated > 1.2-fold; black circles: genes with < 1.2-fold change. **F** Heatmap demonstrating the 5 upregulated transcription factors in HG/HL with APN in HUVEC nucleus. Data were analyzed by two-way analysis of variance. Comparisons between each group were made by post hoc analysis via Tukey test (GraphPad Prism 8.0)

**Fig. 3:**
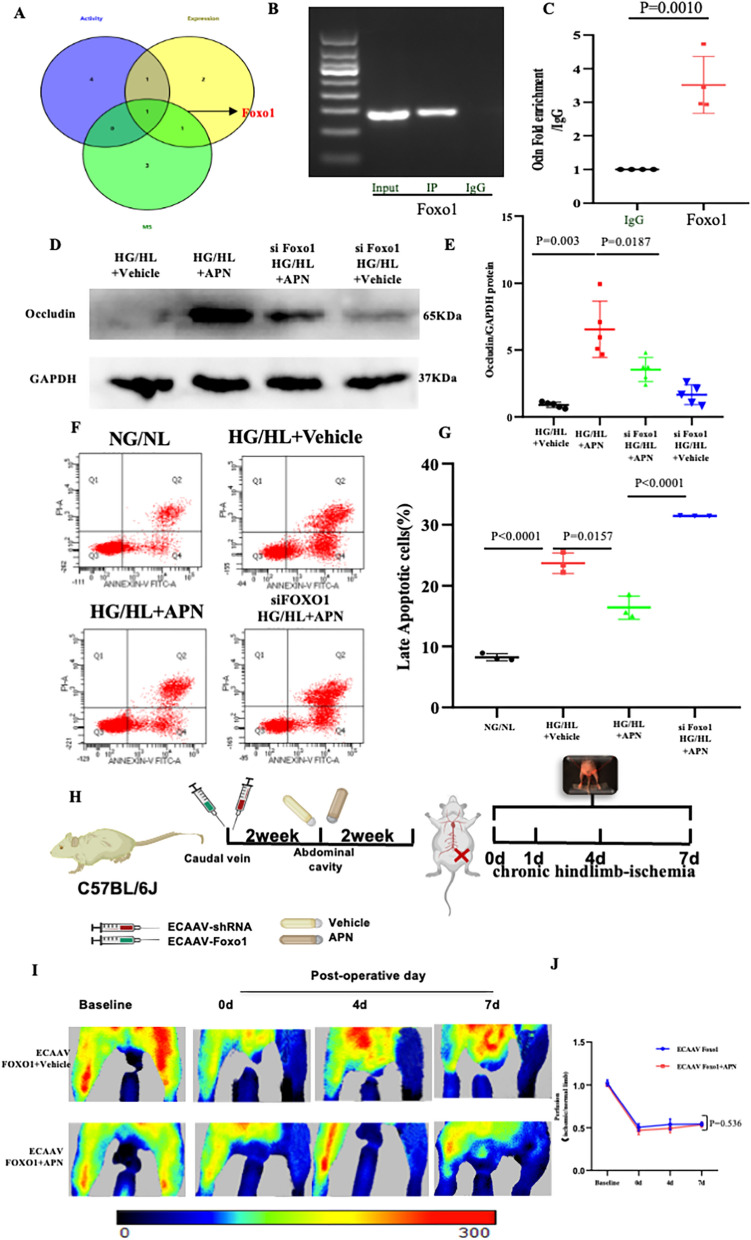
Foxo1 is transcriptional factor of Occludin by APN regulation. **A** Overlapping differential transcriptional factors were identified by Venn map analysis. **B**, **C** CHIP-qPCR to verify the direct interaction between transcriptional factors and Occludin promoters. **D**, **E** APN treatment increased the expression of Occludin. This effect was blocked by Foxo1 knockdown. **F**, **G** APN attenuated high-glucose/high-lipid (HG/HL)–induced endothelial apoptosis in HUVECs. The effect was significantly blunted by Foxo1 knockdown. **H** Schematic of ECAAV-shFoxo1 treatment in adult C57BL/6J mice. APN treatment was performed on two weeks after the ECAAV-shFoxo1 injection. The hindlimb ischemia of mouse were conducted after 2 weeks of APN treatment. **I**, **J** The blood flow of mice subjected to hindlimb ischemia was monitored at indicated time points in blinded fashion. N = 5 independent experiments/group. Data were analyzed by 1-way ANOVA (**C**, **J**) and 2-way ANOVA followed by Tukey test for post hoc analysis (**E**, **G**; GraphPad Prism 8.0)

Having shown that APN treatment increased the mRNA level of Occludin in an APPL1-dependent manner, we endeavored to identify the responsible transcription factors. To obtain the most definitive evidence, we employed three distinct approaches. (1) We utilized a transcription factor activity detection kit to ascertain HG/HL-inhibited, APN-activated, APPL1-mediated transcription factors. As shown in Fig. 2A–C, among 84 transcriptional factors known to be critical in activity detection assay, 29 exhibited a significant downregulation in HG/HL-treated HUVECs. APN treatment resulted in the upregulation of 39 transcriptional factors, 15 of which overlapped with genes exhibiting downregulation in response to HFD. Sixty genes exhibited significant downregulation in HUVECs subjected to HG/HL treatment and transfected with siAPPL1. Venn diagram analysis revealed that APN treatment, in an APPL1-dependent manner, restored the activities of six transcription factors (*SRF, FAST-1, MYB, MYC-MAX, Foxo1*, and *Gfi-1*) that had been downregulated by HFD. (2) RT^2^ Profiler human transcription factor PCR Array was executed to detect the expression of transcription factor. 12 transcription factors were significantly upregulated in HUVECs treated by APN administration, of which 5 overlap with transcription factors downregulated by siAPPL1(*Foxo1, MAX, MYB, SMAD4,* and *HDAC1*) (Fig. 2D–F). (3) Our previous research demonstrated that APN actively promotes the nuclear translocation of APPL1 [16]. Therefore, we investigated the potential impact of APN-mediated, nuclear-translocated APPL1 on the regulation of transcription factors. Using liquid chromatography-mass spectrometry analysis on the nuclear fraction of HUVECs, we identified 5 nuclear transcriptional factors (*HDAC1, Foxo1, BACH2, MGA,* and *HIVEP2*) as potential interactors with APPL1(Table S1). Through Venn overlapping analysis utilizing three distinct methods, we identified Foxo1 as the sole transcription factor whose upregulation by APN treatment is dependent on APPL1 (Fig. 3A). Further investigations revealed a direct binding affinity between APPL1 and Foxo1, with APN significantly enhancing this interaction, as evidenced by co-immunoprecipitation assays (Figure S6). In addition, CHIP-qPCR assay was employed to verify the direct interaction between transcriptional factors and the promoter regions of the *Ocln* gene. The work identified a > threefold enrichment of the Foxo1 mark in the *Ocln* promoter region compared to vehicle group. (Fig. 3B, C). To elucidate the role of transcription factors in APN-induced Occludin expression, we employed RNA interference techniques to diminish the level of Foxo1 in ECs. The data show that in both normal (Figure S7) and HG/HL-induced ECs (Fig. 3D, E), the augmentation of Occludin expression facilitated by APN is impeded by the silencing of the *Foxo1* gene. Taken together, these results suggest that Foxo1 acts as a transcriptional regulator for Occludin, functioning in a manner dependent on APN-mediated APPL1 interactions.

To further understand the pathological and therapeutic implications of APN-mediated transcriptional regulation and its impact on Occludin protein expression, we assessed the effect of Foxo1 knockdown on APN's protective role against apoptosis in ECs induced by HG/HL. Administration of APN markedly attenuated apoptosis in ECs induced by HG/HL conditions. However, this protective effect of APN was nullified by the knockdown of Foxo1 (Fig. 3F, G). In addition, AAV9/shRNA-mediated EC-specific *Foxo1* knockdown mice were constructed (Figure S8), and then animals were subjected to chronic hindlimb-ischemia (Fig. 3H). Our results showed that APN administration failed to improve blood flow recovery in EC-specific *Foxo1* knockdown mice (Fig. 3I, J). The findings indicate that Foxo1 plays a pivotal role in vascular protection mediated by APN.

### APN inhibited the degradation of Occludin by ubiquitin–proteasome pathway (UPP)

**Fig. 4:**
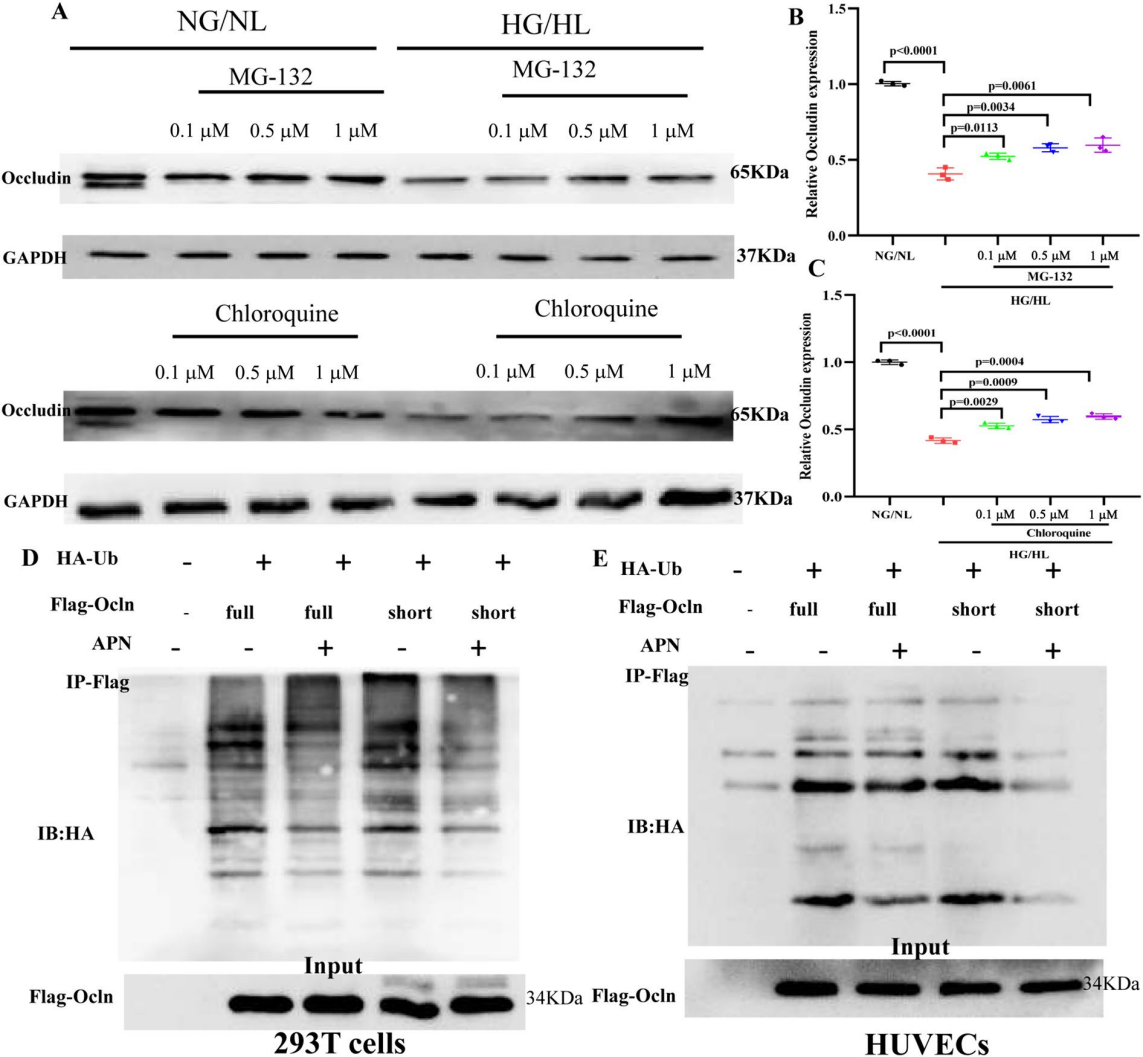
APN inhibited the degradation of Occludin by ubiquitin–proteasome pathway (UPP). **A**–**C** HG/HL promoted Occludin degradation by ubiquitin–proteasome pathway (UPP) and lysosome pathway. **D**, **E** Adiponectin promoted Occludin expression by inhibiting UPP. Data were analyzed by two-way ANOVA followed by Tukey test for post hoc analysis (GraphPad Prism 8.0)

It is well-established that upregulation of protein expression can be achieved through the modulation of its stability, which involves augmenting protein synthesis and impeding degradation [17]. We have already shown in Result 2 that APN enhances Occludin expression by stimulating protein synthesis. The subsequent phase of our investigation will focus on determining whether APN also promotes Occludin expression by inhibiting its degradation. The effect of MG-132 (a proteasome inhibitor) or chloroquine (a lysosome inhibitor) upon HG/HL-induced Occludin downregulation was determined. As shown in Fig. 4A–C, both MG-132 and chloroquine substantially impeded the degradation of Occludin in HG/HL-treated HUVECs. This finding indicates that both lysosomal and proteasomal pathways play pivotal roles in the HG/HL-induced degradation of Occludin.

To further determine whether APN promotes the expression of Occludin through inhibiting Occludin degradation, we conducted the following experiments. (1) To test whether APN promotes the expression of Occludin through inhibiting ubiquitin–proteasome pathway, HEK293T cells were transfected with FLAG-tagged Occludin and HA-tagged ubiquitin (HA-Ub) together with or without APN. As shown in Fig. 4D, ubiquitination of Occludin was detected in the presence of APN. Strikingly, APN significantly reduced ubiquitination levels of Occludin. To confirm these results, we also measured Occludin ubiquitination level in HUVECs with or without APN under HG/HL treatment. Consistently, APN obviously inhibited Occludin ubiquitination. In particular, the inhibitory role of APN is more obvious in HG/HL-induced HUVECs (Fig. 4E). (2) To further substantiate whether the lysosomal pathway is implicated in the regulation of Occludin expression by APN, the colocalization and interaction between LAMP2, a lysosomal marker, and Occludin were assessed using immunofluorescent staining. HG/HL markedly enhanced the colocalization of LAMP2 and Occludin in HUVECs. However, APN did not alter this LAMP2/Occludin colocalization, regardless of whether the cells were cultured under HG/HL conditions or not (Figure S9). These data suggest the proteasome-mediated degradation of Occludin is inhibited under APN-mediated condition.

### Occludin phosphorylation at Tyr467: the crucial locus for APN's suppression of Occludin ubiquitination

Interplay between post-translational modifications like glycosylation and phosphorylation influences protein ubiquitination and degradation [18–20]. In addition, recent study demonstrated that VEGF-induced Occludin phosphorylation is involved in vascular barrier maintenance [21]. Simultaneously, Ser-490 phosphorylation of Occludin is an essential prerequisite for its ubiquitination [22], suggesting that there may be an interaction between Occludin ubiquitination and phosphorylation. Therefore, we hypothesize that APN may increase Occludin expression by promoting its phosphorylation and inhibiting its ubiquitination-mediated degradation. To define the relationship between APN and Occludin phosphorylation, two experiments was performed. Firstly, according to the published paper, a novel method known as manganese phosphate-affinity SDS-PAGE (Mn^2+^-Phos-tag SDS-PAGE) was used to detect the mobility shifts in phosphoproteins [23]. Therefore, in this study, to further investigate whether APN can enhance Occludin phosphorylation, we incubated human recombinant APN and Occludin in a time-dependent manner, both with and without ATP, in a cell-free system. Samples were collected and immunoblotted using phosphate-affinity SDS-PAGE with antibody against Occludin. The band representing Occludin was clearly migrated after 5 min of APN treatment (Figure S10). Secondly, Occludin phosphorylation, detected using an antibody targeting phosphoserine (pSer), was observed under basal conditions. Interestingly, APN failed to elicit a substantial increase in Occludin phosphorylation, in stark contrast to HG/HL, which demonstrated a significant opposing effect (Figure S11). Further empirical validation is required to elucidate the phosphorylation levels of Occludin subsequent to APN exposure. However, the extant data compellingly indicate that APN plays a contributory role in the phosphorylation process of Occludin.

**Fig. 5:**
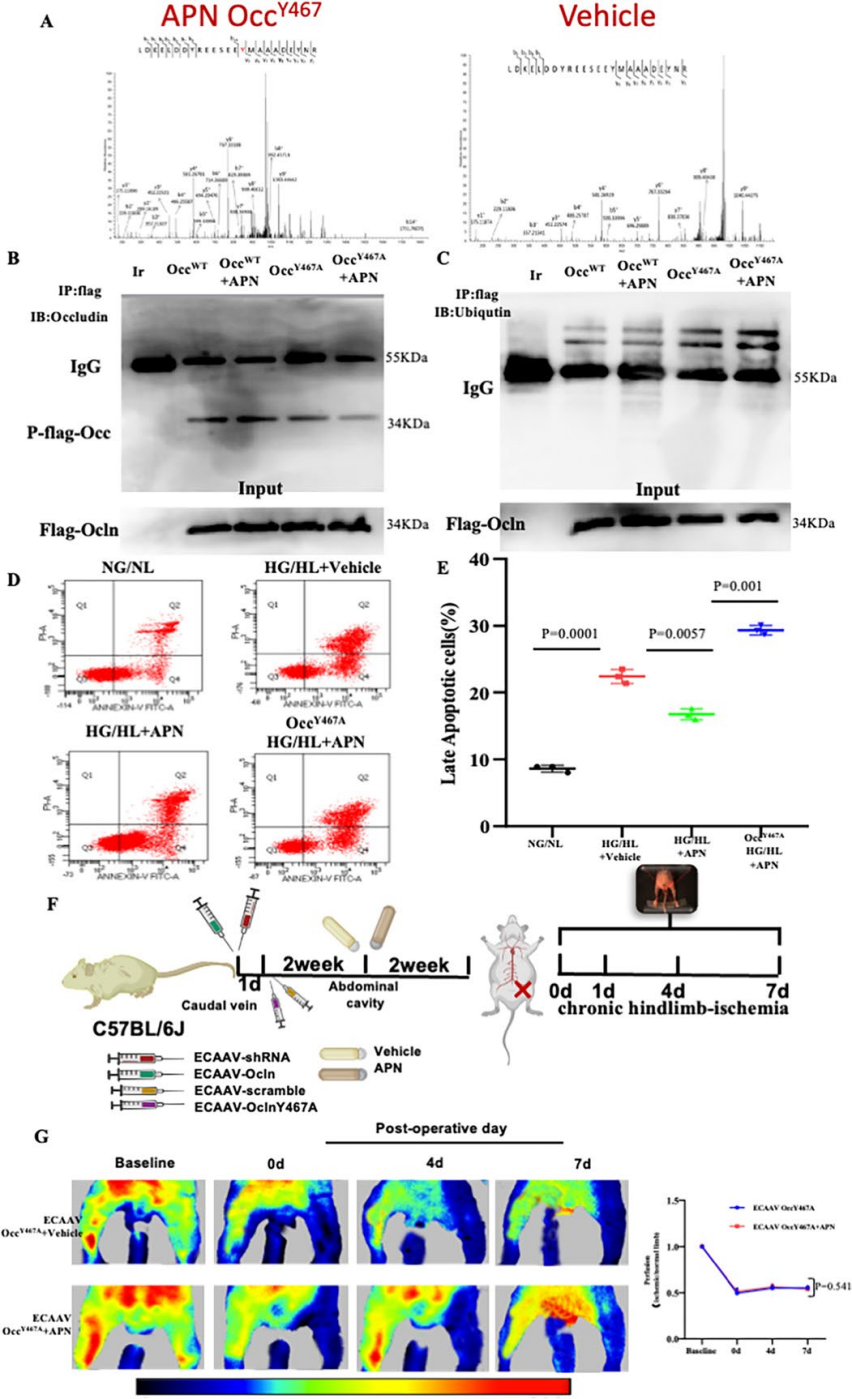
APN suppressed Occludin ubiquitination by phosphorylating Tyr467 on Occludin. **A** MS analysis identified phosphorylation site that are sensitive to APN. **B** Immunoprecipitation identified APN promoted the phosphorylation of Occludin Y467 site. **C** APN-induced Occludin phosphorylation at tyrosine467 is responsible for Occludin ubiquitination in the endothelium. **D** Apoptosis was measured by annexin V-FITC/PI staining and analyzed with flow cytometry. **E** Occludin phosphorylation is involved in APN-mediated anti-apoptotic effects. **F** Schematic of mouse model construction. There is a one-day interval between caudal vein injection of ECAAV-sh*Ocln* and ECAAV-*Ocln* Y467A. **G** The blood flow of mice subjected to hindlimb ischemia was monitored at indicated time points in blinded fashion. N = 6–8/group. Data were analyzed by two-way ANOVA followed by Tukey test for post hoc analysis (GraphPad Prism 8.0)

To identify the specific site(s) responsible for APN-induced Occludin phosphorylation that inhibits it ubiquitination, mass spectrometry was conducted. For immunoblotting purpose, conventional SDS-PAGE methodology was employed. Following the electrophoresis process, the gels were stained with Coomassie Brilliant Blue for visualization. Protein bands demonstrating differential expression profiles in response to a 5-min incubation with or without APN were meticulously analyzed using mass spectrometry (MS). This in-depth MS analysis elucidated that the Tyr467 site exhibits sensitivity to APN modulation (Fig. 5A). Next, we determined the significance of Tyr467 phosphorylation modification by replacing Tyr467 with alanine (OccludinY467A). HEK293T cells were transfected with vectors expressing either Flag-Occludin WT or Flag-Occludin Y467A with or without APN. Cells were collected, homogenized, and the resulting samples underwent immunoprecipitation with an anti-flag antibody, followed by immunoblotting with an anti-phosphotyrosine (p-Tyr) antibody. APN failed to promote the phosphorylation of Occludin after reexpressing a mutated Occludin in HEK293T cells (Fig. 5B).

To elucidate the interplay between Occludin Y467 phosphorylation and ubiquitination following APN treatment, co-immunoprecipitation experiments were conducted. Samples underwent immunoprecipitation using an antibody against Flag, followed by immunoblotting with an antibody targeting ubiquitin. Immunoblot analyses revealed that the APN-mediated inhibition of Occludin ubiquitination was abrogated upon transfection with OccludinY467A plasmids (Fig. 5C). Consequently, it is evident that APN-induced phosphorylation of Occludin at Tyrosine 467 plays a pivotal role in regulating Occludin ubiquitination within endothelial cells.

To ascertain the functional significance of APN-mediated post-translational modifications and consequent protein expression changes of Occludin, we investigated the impact of reintroducing a mutated form of Occludin on APN's protective effect against apoptosis induced by HG/HL in ECs. As shown in Fig. 5D, E, APN treatment markedly reduced apoptosis in ECs induced by HG/HL exposure. Nevertheless, in the context of HG/HL-treated ECs reexpressing a mutated form of Occludin, APN did not exhibit the same inhibitory effect on endothelial apoptosis. Simultaneously, it was observed that the administration of APN did not contribute to the amelioration of blood flow recovery in a mouse model characterized by endothelial cell-specific reexpression of a mutated form of Occludin (Fig. 5F, G). In all, these results indicate that adiponectin promotes Occludin phosphorylation, suppresses the degradation of Occludin, and increases Occludinn expression, protecting ECs. Collectively, these findings suggest that APN facilitates the phosphorylation of Occludin, inhibits its degradation, and enhances Occludin expression, thereby contributing to the protection of ECs.

### Elevated plasma Occludin levels in the context of diabetes

**Fig. 6:**
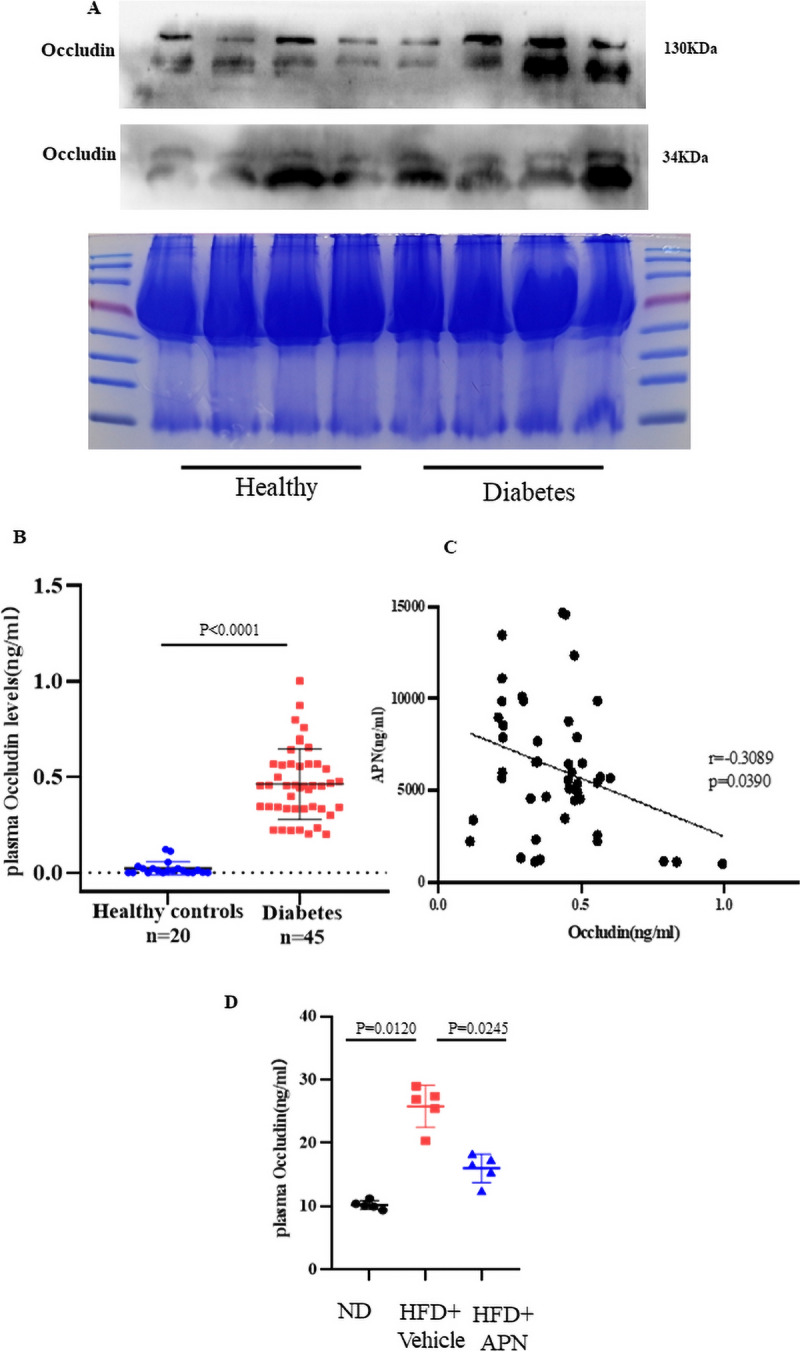
The plasma levels of Occludin in diabetes. **A** The western blot analysis of Occludin protein levels in the plasma of individuals with diabetes and healthy controls. **B** The plasma levels of Occludin in individual measured by ELISA. **C** Plasma Occludin concentrations were negatively correlated with APN (n = 65, r = 0.3089, p = 0.0390). **D** The plasma concentrations of Occludin in mice assessed by ELISA. Comparisons between each group were made by post hoc analysis via Tukey test (GraphPad Prism 8.0)

A recent study has shown that serum Occludin can serve as a biomarker [24]. Therefore, detecting changes in serum Occludin levels may help indicate the occurrence and progression of these diseases. For a comprehensive analysis of plasma Occludin levels in individuals with diabetes, a series of systematic experiments were conducted. A total of 65 subjects participated in this study, comprising 45 with Type 2 Diabetes Mellitus (T2DM) and 20 controls. Demographic information along with baseline clinical and biochemical characteristics are detailed in Table S1. Statistical analysis revealed elevated fasting blood glucose (FBG) levels. Firstly, we identified elevated plasma Occludin levels in diabetes through Western blot analysis (Fig. 6A). Secondly, a quantitative comparison demonstrated a significant increase in plasma Occludin levels within the diabetic group as compared to the healthy control group (0.4961 ng/mL [IQR, 0.1656–0.7726] versus 0.0132 ng/mL [IQR, 0.000–0.03342]; p < 0.01, Fig. 6B). Employing two methodologies, both consistently revealed heightened plasma Occludin concentrations in diabetic patients compared to their healthy counterparts. Additionally, univariate logistic regression analysis indicated a negative correlation between plasma Occludin levels in the Type 2 Diabetes Mellitus (T2DM) group and APN (Fig. 6C). In addition, we conducted an ELISA assay to quantify plasma Occludin levels in diabetic mice treated with APN. The results demonstrated that plasma Occludin levels were elevated in diabetic mice, while adiponectin treatment effectively reduced these elevated levels (Fig. 6D).

## Discussion

In the present study, we provide evidence that APN plays a critical role in vascular endothelial protection induced by HG/HL in an Occludin-dependent manner. Mechanistic investigations reveal that Foxo1 acts as a key transcription factor for Occludin, regulated by APN. Concurrently, our findings also demonstrate that APN impedes Occludin degradation through the ubiquitin–proteasome pathway (UPP). Notably, phosphorylation at the Tyr467 site on Occludin emerges as the pivotal mechanism underlying APN's inhibitory effect on Occludin ubiquitination. Our findings suggest that APN induces Occludin expression both through transcriptional regulation and post-translational modifications, thereby exerting protective effects against diabetic vascular endothelial injury (*Graphic Abstract*). Furthermore, this study is the first to demonstrate a significant elevation in plasma Occludin levels in the context of diabetes.

Occludin, a prominent tight junction protein, features four transmembrane segments and two extracellular loops. The first loop is characterized by a high concentration of tyrosine and glycine, while the second loop is also rich in tyrosine. Additionally, it has two intracellular domains: an NH_2_-terminal cytoplasmic domain and a COOH-terminal cytoplasmic domain [11]. Occludin is expressed across multiple species, including humans, rats, and mice [11, 12]. It is predominantly found in the endothelial cells of arteries and veins, as well as in crucial barriers such as the blood–brain barrier and the blood-retinal barrier. Changes in the expression of Occludin are crucially linked to the dysfunction of vascular endothelial cells [25]. In diabetic retinal vascular endothelial cells, the expression of Occludin is diminished, which contributes to vascular dysfunction characterized by increased vascular permeability, impaired neovascularization, and elevated inflammatory responses [13]. This indicates that reduced Occludin levels may be a significant factor in the vascular dysfunction observed in diabetes. Liu et al. isolated primary mouse retinal endothelial cells for in vitro culture and found that Occludin S490 phosphorylation is a crucial factor for retinal endothelial cell tube formation, as well as for cell proliferation and migration [26]. In addition, in a rat model of cerebral ischemia, Occludin expression in the blood–brain barrier was observed to initially increase at 24 h and subsequently decrease by 72 h [27]. In this study, we demonstrate that APN significantly upregulates the transcriptional and translational levels of Occludin, a process that is mediated via the APPL1 pathway, as evidenced by our in vivo and in vitro investigations. To rigorously ascertain Occludin's role in diabetic vascular endothelial injury, we utilized two distinct animal models: mice with endothelial cell-specific Occludin knockdown mediated by AAV9/shRNA, and a broader AAV9/shRNA-mediated Occludin knockdown model. These models were maintained under diabetic conditions to validate the phenotype. Our findings indicate that adiponectin mitigates endothelial apoptosis and fosters angiogenesis in an Occludin-dependent manner. Collectively, we provide clear evidence that Occludin may represent a novel therapeutic target for combating diabetic vascular injury.

It is well-established that the dynamics of gene and protein expression are governed by an array of factors, including transcriptional regulatory mechanisms, targeted protein degradation pathways, and various epigenetic processes [28]. The dynamic transcriptional process is typically orchestrated by transcription factors (TFs), which selectively bind to upstream regions of target genes known as promoters. This binding event serves to either activate or repress the synthesis of RNA molecules, subsequently influencing the downstream production of proteins [29, 30]. Simultaneously, our results demonstrated that the mRNA level of Occludin was inhibited in endothelial cells treated by HG/HL and promoted by APN treatment in an APPL1-dependent manner. These findings tentatively indicate that the transcriptional regulation of Occludin may be compromised or hindered within the milieu of diabetic vascular injury. Nonetheless, it is plausible that APN could exert a facilitative role in this process, thereby contributing to the modulation of Occludin expression. In this study, assays measuring transcription factor expression and activity, combined with advanced mass spectrometry techniques, were methodically employed to identify the specific transcription factors involved in orchestrating the APN/APPL1 signaling cascade. Previous research from our lab has demonstrated that APN promotes APPL1 nuclear translocation and regulates the vasculoprotective gene expression, suggesting that dysregulation of APPL1-mediated epigenetic regulation represents novel mechanisms leading to diabetes-induced pathological vascular remodeling [16]. In this study, we observed that APN facilitates the interaction between APPL1 and Foxo1. Moreover, extensive analysis identified Foxo1 as a regulatory factor in the modulation of Occludin expression. This discovery has been substantiated through ChIP-qPCR validation. Western blot analysis delineated that the augmentation of Occludin protein levels induced by APN was nullified subsequent to the knockdown of Foxo1. Functionally, the silencing of Foxo1 effectively negated the protective effects of APN against HG/HL-induced apoptosis and angiogenesis, as evidenced in both in vitro and in vivo models. These findings suggest that APN regulates the expression of Occludin, thereby imparting protective effects on the vascular endothelium in the diabetic context. This modulation is facilitated through the involvement of the transcription factor Foxo1.

Protein expression can be upregulated via two fundamental pathways: the first involves the stimulation of protein biosynthesis, while the second involves the suppression of proteolytic degradation [31]. This research has demonstrated that APN enhances the biosynthesis of Occludin via transcriptional regulatory mechanisms. Nonetheless, it remains uncertain whether APN also plays a role in mitigating the proteolytic degradation of Occludin. The two major intracellular degradation system in eukaryotic cells are the ubiquitin–proteasome system (UPS) and autophagy lysosome pathway (ALP) [32]. The ALP pathway involves soluble proteins with specific amino acid sequences being recognized and bound by molecular chaperones. Subsequently, these proteins are transported into lysosomes through the lysosomal-associated membrane protein 2a (LAMP2A) receptor located on the lysosomal membranes. Within the lysosomes, these proteins undergo degradation by hydrolases [33]. In our study, we found that HG/HL-induced lysosomal-mediated degradation of Occludin is independent on APN-mediated condition. UPS plays a crucial role in eukaryotic cells, mediating 80–85% of protein degradation [34]. Notably, it has been confirmed that Occludin, a significant protein, is subject to ubiquitination within this system [35]. Liu and Lee et al. indicated that the degradation of Occludin is linked to Itch and UBC-4, a ubiquitin-conjugating enzyme. This leads to the ubiquitination of Occludin, disrupting the tight junctions in the blood-testosterone barrier [36]. Our research team has been the first to demonstrate that APN inhibits the degradation of Occludin through the Ubiquitin–Proteasome Pathway (UPP), revealing a novel regulatory mechanism in protein turnover. Moreover, previous studies have indicated that phosphorylation often serves as a priming event for ubiquitination [37]. This process, in turn, influences protein phosphorylation by modulating the activities of kinases. Specific phosphorylation events are known to regulate other post-translational modifications, such as ubiquitination and sumoylation [38]. This interplay can significantly influence the stability of the protein and its rate of degradation, underscoring the intricate mechanisms governing protein turnover [39, 40]. It has been demonstrated that phosphorylation at Ser490 facilitates the subsequent ubiquitination and endocytosis of Occludin, leading to disruptions in the tight junctions [22]. However, in this study, we observed that the APN-induced phosphorylation of Occludin does not occur at serine residues. Instead, we identified a novel phosphorylation site on Occludin Tyr467. Phosphorylation at this site exhibits effects opposite to those observed at the previously identified serine phosphorylation sites. In addition, we determined that Occludin Tyr467 phosphorylation acts as the responsible site for the inhibitory role of APN to Occludin ubiquitination. Finally, endothelial-specific reexpressing a mutated Occludin upon APN’s protective effect against HG/HL-induced ECs apoptosis and inhibited angiogenesis was determined in mouse model. The results determined that APN-induced Occludin phosphorylation at Tyrosine467 is responsible for HG/HL-induced apoptosis and angiogenesis in the vascular endothelium.

The conventional perspective posits that Occludin, as an integral membrane protein situated at tight junctions, is primarily responsible for upholding cellular permeability. However, recent advancements in basic medical research have revealed that proteolytically cleaved fragments of Occludin, once released into the circulatory system, show a significant and notable correlation with the integrity of the blood–brain barrier [25]. These findings illuminate new aspects of Occludin's role beyond its traditional functions. In addition, Occludin serve as a potential biomarker to predict the severity of acute ischemic stroke, hemorrhagic transformation, and patient prognosis. Meanwhile, it has been reported that Occludin undergoes hydrolyzation by metalloproteinases and caspases, subsequently allowing its entry into the bloodstream [41]. These results suggested that the degradation of Occludin may be involved in the occurrence and development of many diseases. In this study, plasma samples were obtained from diabetic patients as well as healthy individuals, with the aim of quantifying the levels of Occludin in these samples. The data unequivocally demonstrates elevated serum levels of Occludin, accompanied by observable fragmentation patterns, within the context of diabetes, as evidenced by Western blot analysis. Finally, we found that plasma Occludin levels were increased in patients with diabetes compared to the healthy group, and Occludin concentration was negatively correlated with APN level in the setting of diabetes. Collectively, we provide clear evidence that Occludin may represent a novel therapeutic approach against the diabetic vascular inflammatory effects.

This study elucidates a novel mechanism underlying the vascular protective effects mediated by APN. We discovered that APN-mediated endothelial protection involves both the promotion of Occludin expression through transcription factor activation and the inhibition of Occludin degradation. These findings suggest that Occludin serves as a pivotal mediator in adiponectin-mediated vascular protection in diabetes. Conversely, the reduction in Occludin levels hinders the vascular protective effects of APN.

## Supplementary Information

Supplementary Material 1.

## Data Availability

The authors declare that all the data supporting the findings of this study are available within the article and its Supplemental information files. All materials in this article are available upon reasonable request from the corresponding authors.
