# Supplementary material for: Transcription and post-translational mechanisms: dual regulation of adiponectin-mediated Occludin expression in diabetes
Source: Cell Biosci. 2024 Oct 1;14:126. doi: 10.1186/s13578-024-01306-5 (PMC11443667; doi:10.1186/s13578-024-01306-5)
Supplement: Supplementary file 1 — Supplementary Material 1. [file 13578_2024_1306_MOESM1_ESM.docx]

SUPPLEMENTAL INFORMATION

**Supplemental Figures and Figure Legends**


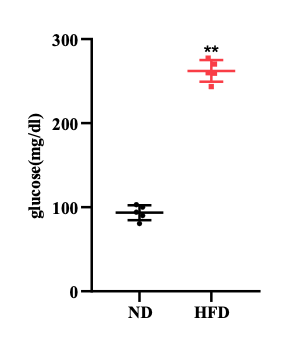


**Supplemental Figure 1. Type 2 diabetic mice model.** WT mice were fed with HFD for 12 weeks to induce type 2 diabetes mice model. The fasting glucose level (Data are represented as mean±SEM.N=5~8, ^**^*P*<0.01 vs ND). ND: normal diet; HFD: high fat diet


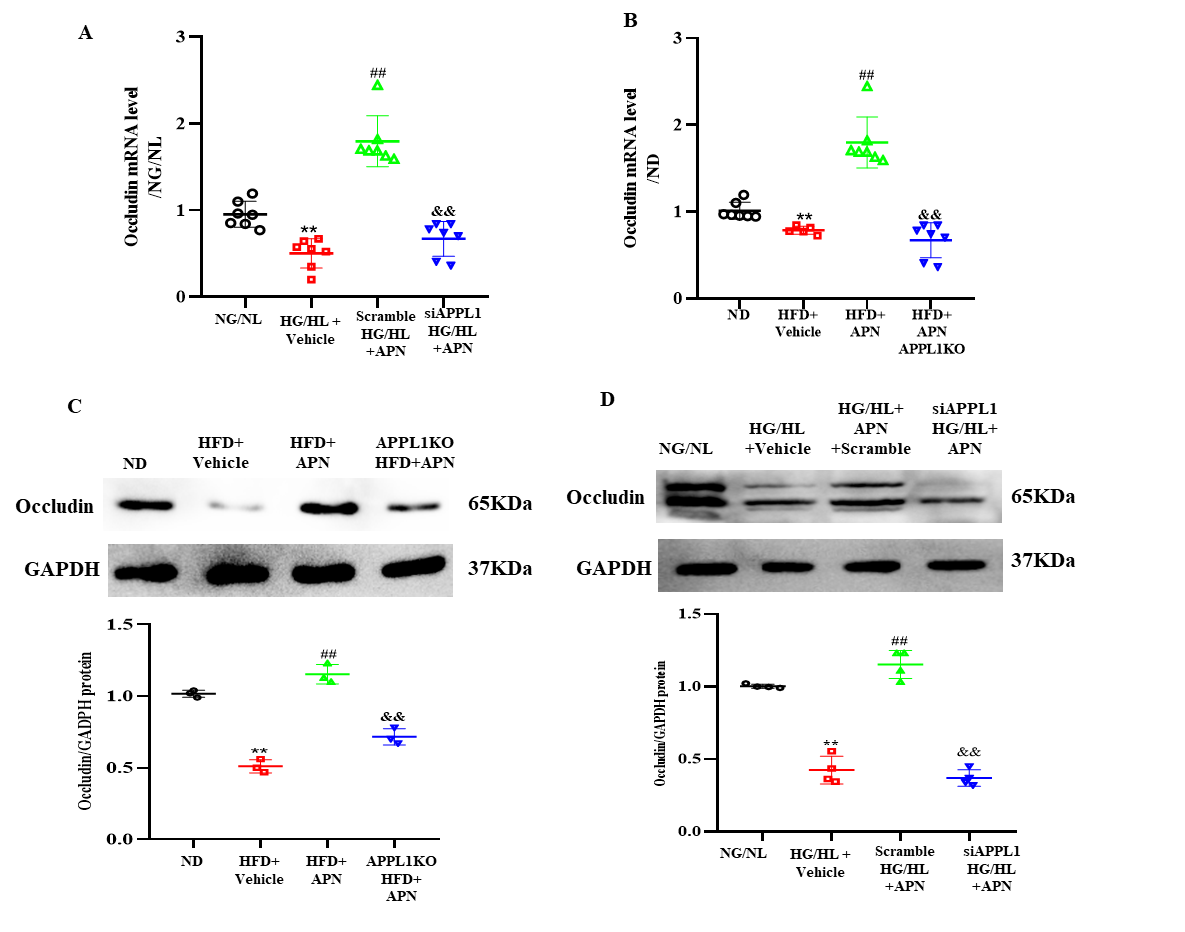


**Supplemental Figure2. The expression of Occludin in HG/HL-induced, APN-stimulated and APPL1-influenced condition in vivo and in vitro. A**. The mRNA levels of Occludin in HUVECs (N=7, ** *P* <0.01 vs. NG/NL; ^##^*P* <0.01 vs. HG/HL+vehicle; ^&&^*P* <0.01 vs. Scramble+HG/HL+APN). **B**. The mRNA levels of Occludin in WT or APPL1KO mice (N=7, ** *P* <0.01 vs. ND; ^##^*P* <0.01 vs. HFD+vehicle; ^&&^*P* <0.01 vs. HFD+APN). **C**. The expression of Occludin in WT or APPL1KO mice (N=3, ** *P* <0.01 vs. ND; ^##^*P* <0.01 vs. HFD+vehicle; ^&&^*P* <0.01 vs. HFD+APN). **D**. The expression of Occludin in HUVECs (N=4, ** *P* <0.01 vs. NG/NL; ^##^*P* <0.01 vs. HG/HL+vehicle; ^&&^*P* <0.01 vs. Scramble+HG/HL+APN). Data are represented as mean±SEM.


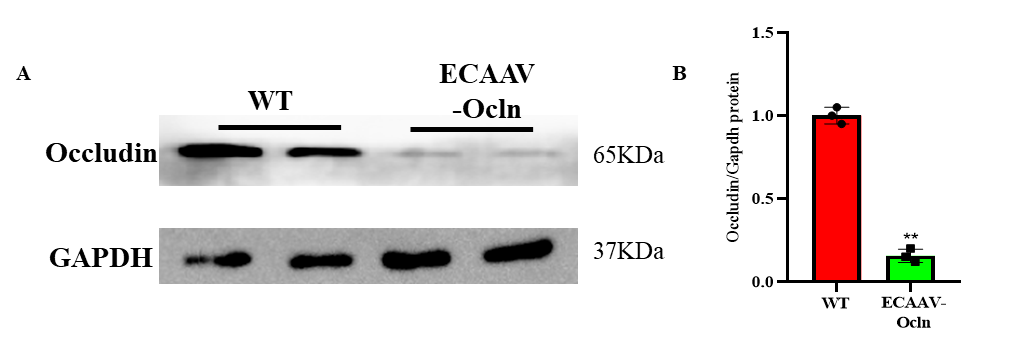


**Supplemental Figure3.** **Western blot examined the expression of Occludin in aortic vessels of mice.** (Data are represented as mean±SEM. N=3, ^**^*P*<0.01 vs WT).


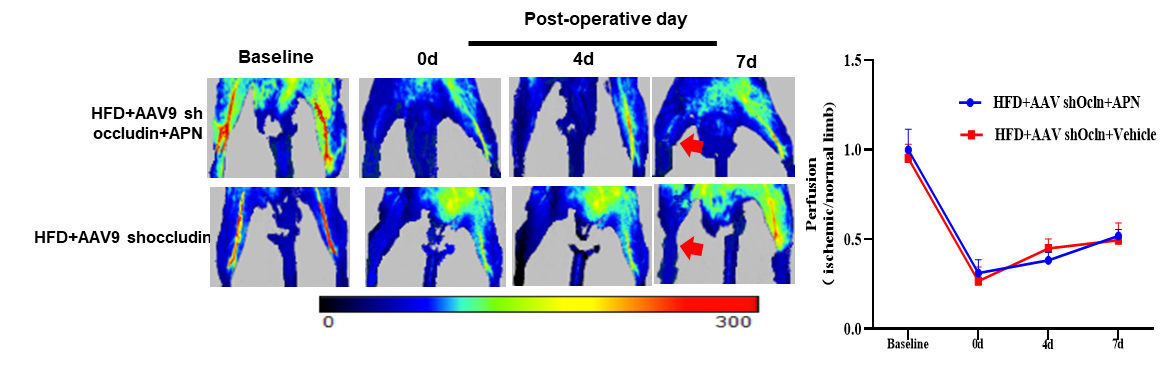

**Supplemental Figure4. The blood flow of mice subjected to hindlimb ischemia was monitored at indicated time points in blinded fashion**. N=6-8/group.


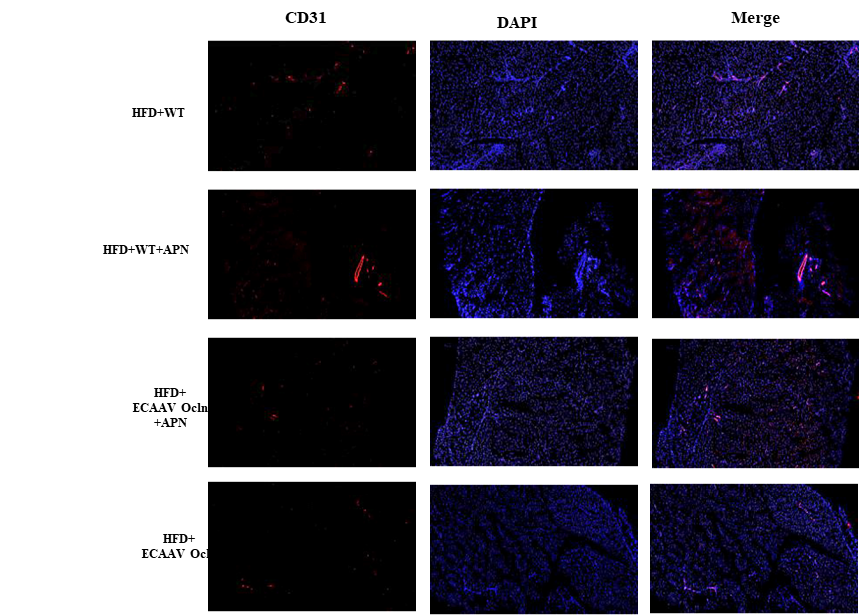


50μm

50μm

50μm

50μm

50μm

50μm

50μm

50μm

50μm

50μm

50μm

50μm

**Supplemental Figure5. Representative images of CD31 immunostaining in mouse limb localization.** **(scale bar=50 µm). Red: CD31.**


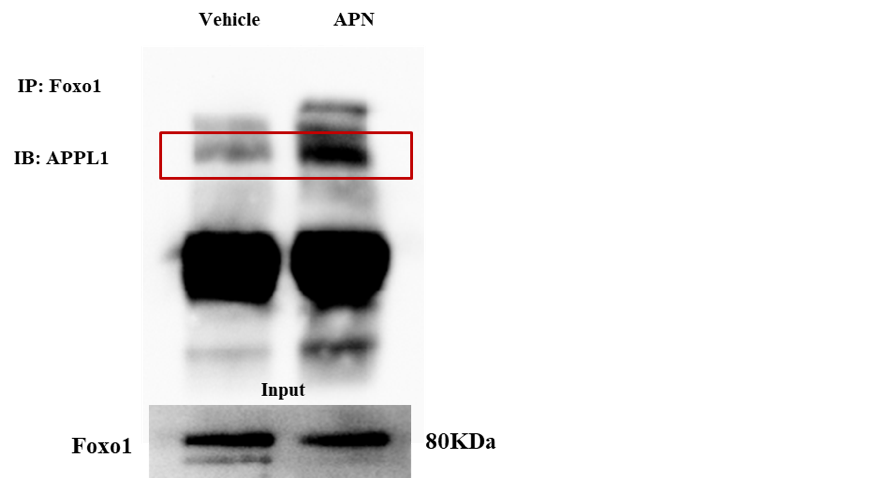


**Supplemental Figure6. APN significantly increased APPL1/Foxo1 interaction in HUVECs**. IB indicates immunoblotting; and IP, immunoprecipitation.


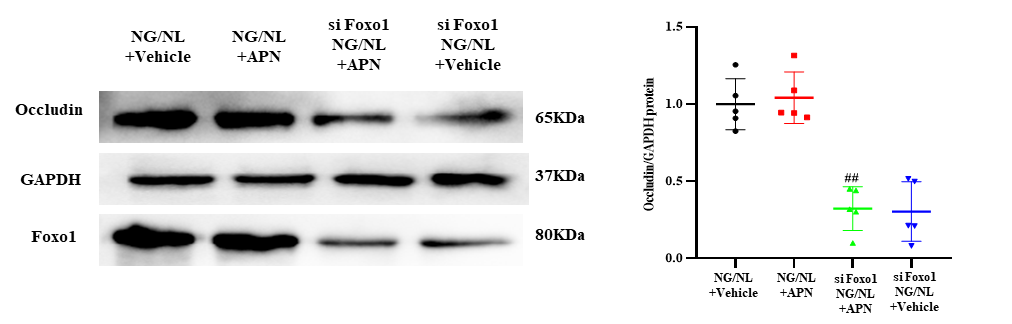


**Supplemental Figure7. The expression of Occludin in knockdown of Foxo1 in NG/NL HUVECs.** (Data are represented as mean±SEM. N=5, ^##^*P＜*0.01 vs NG/NL+APN)


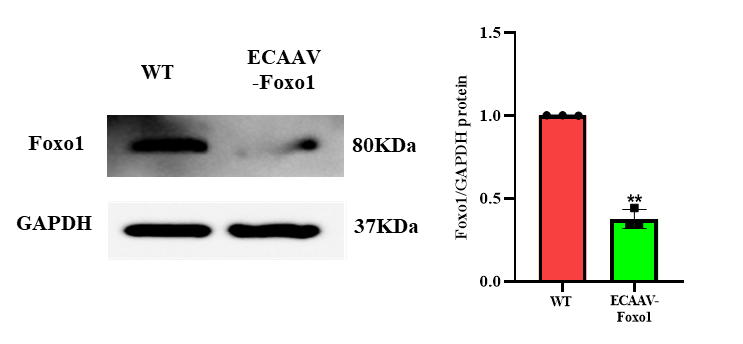


**Supplemental Figure8.** **Western blot examined the expression of Foxo1 in aorta of mice.** (Data are represented as mean±SEM. N=3, ^##^*P＜*0.01 vs WT)


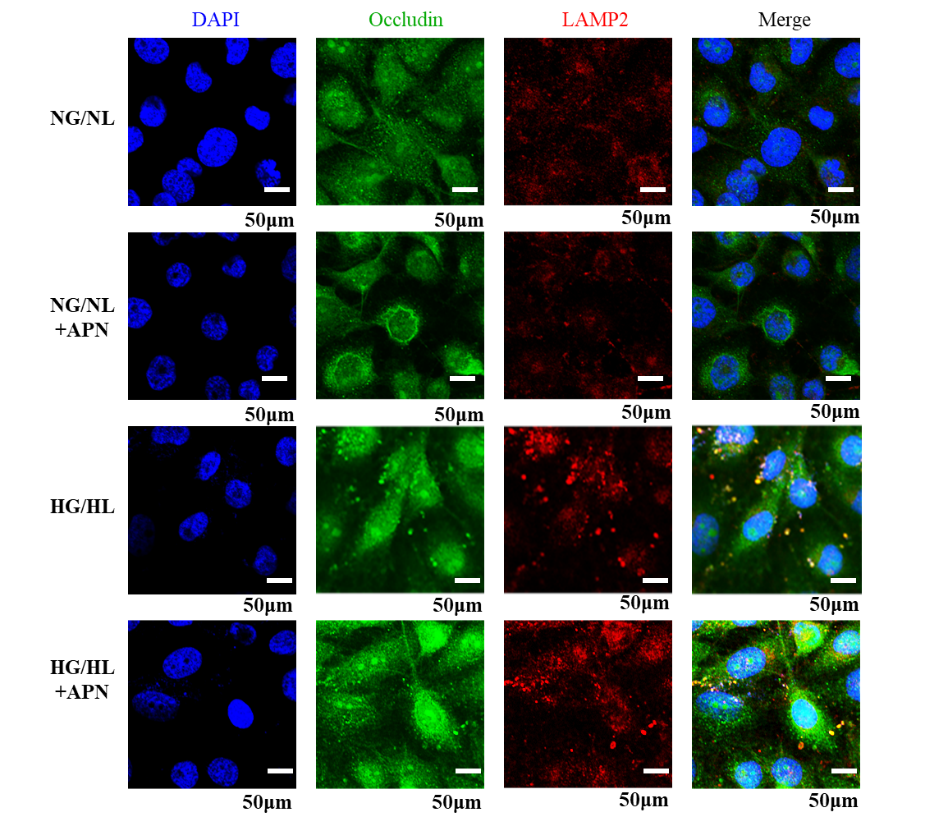


**Supplemental Figure9. Confocal microscopy demonstrating Adiponectin cannot inhibit the combination between Occludin and LAMP2.** (scale bar=50 µm). Green: Occludin; Red: LAMP2.

**
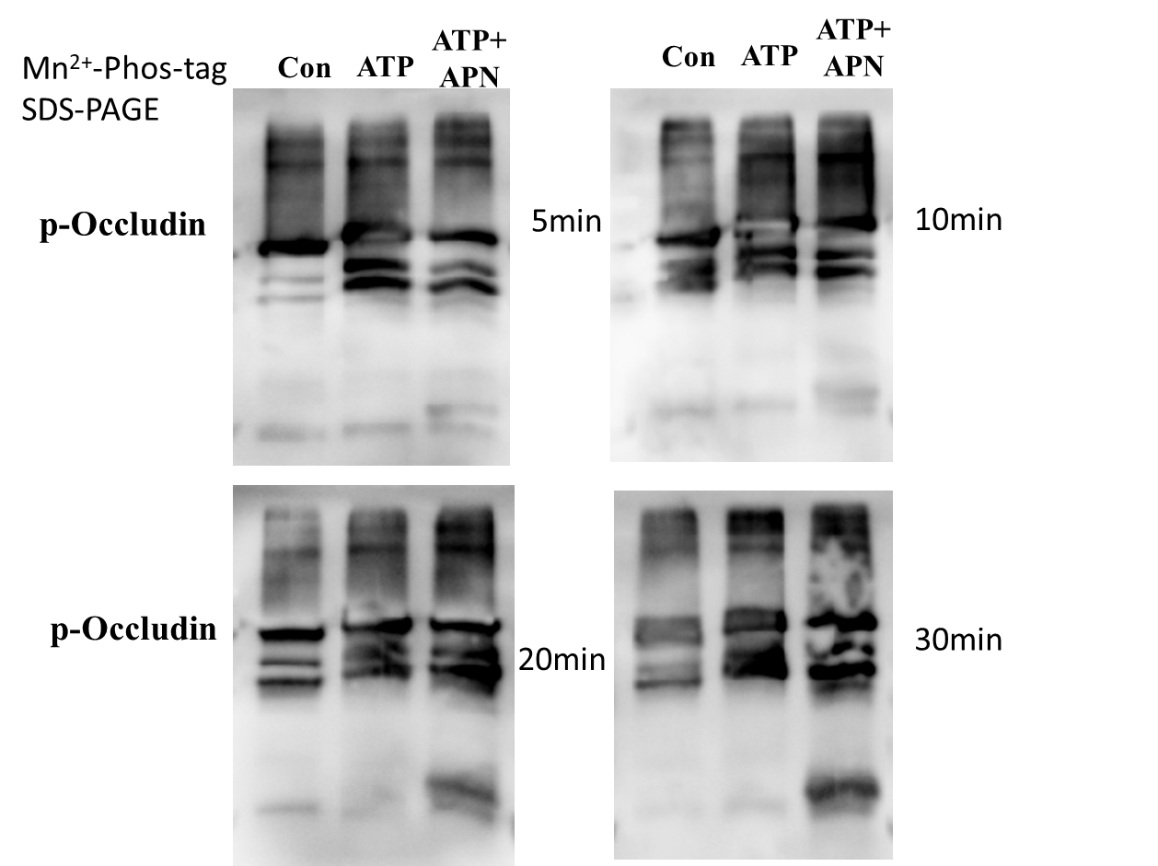
**

**Supplemental Figure10. Western blots analysis represent Occludin was migrated after APN treatment using phosphate-affinity SDS-PAGE with antibody against Occludin.**

**
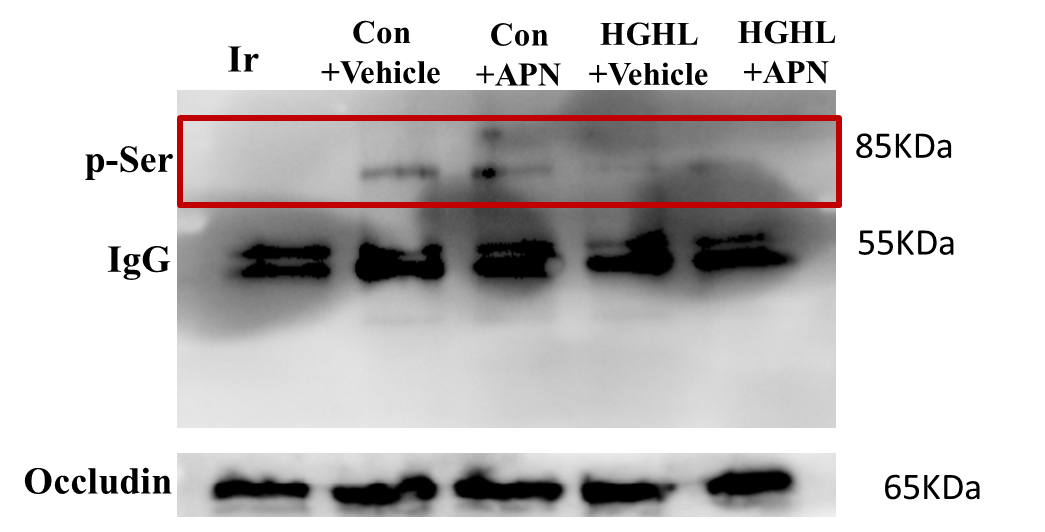
**

**Supplemental Figure11.** Adiponectin had no significant effect on Occludin phosphorylation by immunoprecipitation.

**Supplemental Table 1. The baseline characteristics of the patients and controls**

|  | T2DM (n=45) | Control (n=20) | *P* |
| --- | --- | --- | --- |
| Age (years) | 61(54-68) | 59.5(49.75-69.25) | 0.123 |
| Male (n, %) | 28/17 | 12/8 | 0.078 |
| BMI (kg/m^2^) | 23.50(20.90-26.25) | 24.39(22.65-28.24) | 0.114 |
| SBP (mmHg) | 128(116-141) | 119 (114-129) | 0.113 |
| DBP (mmHg) | 74(70-80) | 75(70-80.75) | 0.726 |
| FPG (mmol/L) | 7.73±2.66 | 5.55±0.56 | ＜0.001 |
| TG (mmol/L) | 1.61±1.49 | 1.47±0.62 | 0.151 |
| TC (mmol/L) | 4.19(3.41-5.31) | 4.20 (3.42-5.19) | 0.565 |
| LDL-C (mmol/L) | 2.47±1.04 | 2.58±0.87 | 0.665 |
| HDL-C (mmol/L) | 1.21±0.45 | 1.31±0.84 | 0.529 |
| hs-CRP (mg/L) | 2.54±2.68 | 2.67±4.44 | 0.456 |
| Plasma APN (ng/ml) | 5134.125±2413.574 | 8022.134±3656.78 | <0.001 |
| Plasma Occludin (ng/ml) | 0.4691±0.3035 | 0.0132±0.01671 | <0.001 |

***Abbreviations:*** BMI: Body Mass Index; SBP: Systolic Blood Pressure; DBP: Diastolic Blood Pressure; FPG: Fasting Blood Glucose; TG: Triglyceride; TC: Total Cholesterol; LDL-C: low-density lipoprotein; HDL-C: high-density lipoprotein; hs-CRP: high-sensitivity C-reactive protein.

**Supplemental Table 2. APPL1 interacting transcriptional factors identified by MS**

| **Gene name** | **Protein Description** | **Mol. Weight (kDa)** |
| --- | --- | --- |
| HDAC1 | Histone deacetylase 1 | 54.74 |
| FOXO1 | Forkhead box protein O1 | 69.51 |
| BACH2 | Transcription regulator protein BACH2 | 94.64 |
| MGA | MAX gene-associated protein | 127.97 |
| HIVEP2 | Transcription factor HIVEP2 | 271.11 |
